# Volumetric Growth and Growth Curve Analysis of Residual Intracranial Meningioma

**DOI:** 10.1227/neu.0000000000002268

**Published:** 2022-12-14

**Authors:** Conor S. Gillespie, George E. Richardson, Mohammad A. Mustafa, Basel A. Taweel, Ali Bakhsh, Siddhant Kumar, Sumirat M. Keshwara, Abdurrahman I. Islim, Shaveta Mehta, Christopher P. Millward, Andrew R. Brodbelt, Samantha J. Mills, Michael D. Jenkinson

**Affiliations:** * Institute of Systems, Molecular and Integrative Biology, University of Liverpool, Liverpool, UK;; ‡ Department of Neurosurgery, The Walton Centre NHS Foundation Trust, Liverpool, UK;; § Department of Oncology, Clatterbridge Cancer Centre NHS Foundation Trust, Liverpool, UK;; ‖ Department of Neuroradiology, The Walton Centre NHS Foundation Trust, Liverpool, UK

**Keywords:** Growth, Meningioma, RANO, Residual, Simpson grade, Surgery, Volume

## Abstract

After meningioma surgery, approximately 1 in 3 patients will have residual tumor that requires ongoing imaging surveillance. The precise volumetric growth rates of these tumors are unknown.

**OBJECTIVE:**

To identify the volumetric growth rates of residual meningioma, growth trajectory, and factors associated with progression.

**METHODS:**

Patients with residual meningioma identified at a tertiary neurosurgery center between 2004 and 2020 were retrospectively reviewed. Tumor volume was measured using manual segmentation, after surgery and at every follow-up MRI scan. Growth rates were ascertained using a linear mixed-effects model and nonlinear regression analysis of growth trajectories. Progression was defined according to the Response Assessment in Neuro-Oncology (RANO) criteria (40% volume increase).

**RESULTS:**

There were 236 patients with residual meningioma. One hundred and thirty-two patients (56.0%) progressed according to the RANO criteria, with 86 patients being conservatively managed (65.2%) after progression. Thirteen patients (5.5%) developed clinical progression. Over a median follow-up of 5.3 years (interquartile range, 3.5–8.6 years), the absolute growth rate was 0.11 cm^3^ per year and the relative growth rate 4.3% per year. Factors associated with residual meningioma progression in multivariable Cox regression analysis were skull base location (hazard ratio [HR] 1.60, 95% CI 1.02–2.50) and increasing Ki-67 index (HR 3.43, 95% CI 1.19–9.90). Most meningioma exhibited exponential and logistic growth patterns (median R^2^ value 0.84, 95% CI 0.60–0.90).

**CONCLUSION:**

Absolute and relative growth rates of residual meningioma are low, but most meet the RANO criteria for progression. Location and Ki-67 index can be used to stratify adjuvant treatment and surveillance paradigms.

## Graphical abstract

**Figure:**
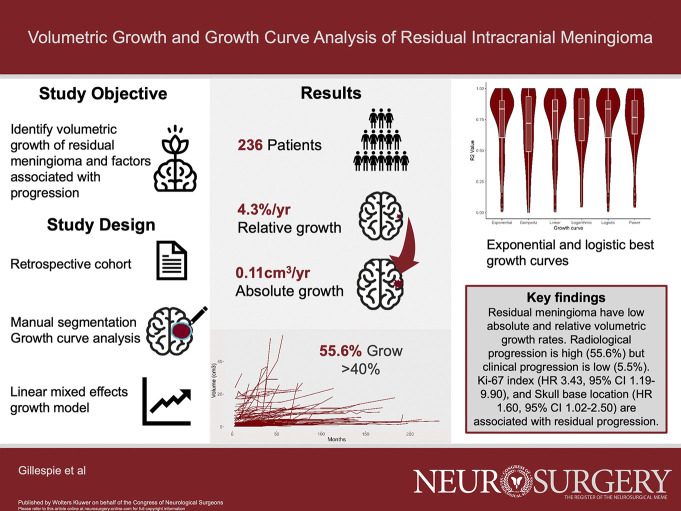

**ABBREVIATIONS:**
EANO: European Association of Neuro-Oncology
EOR: extent of resection
FRT: fractionated radiotherapy
GTR: gross total resection
HR: hazard ratio
ICOM: International Consortium on Meningioma
OS: overall survival
PACS: Picture Archiving and Communications System
PET-CT: Positron Emission Tomography-Computed Tomography
PFS: progression-free survival
SRS: stereotactic radiosurgery
STR: subtotal resection
WHO: World Health Organization.

Meningioma is the most common primary intracranial tumor.^1^ The reported incidence has increased with the wide availability of MRI scanning.^2^ Meningiomas that are symptomatic, increasing in size, or threatening neurovascular structures often require surgery.^3^ Residual tumor remains in up to 33% of meningioma undergoing surgery,^4,5^ identified by the operating surgeon as a Simpson grade IV or V resection,^6^ or on postoperative imaging. The rate of residual meningioma is even higher in some selected series such as those describing skull base meningioma^7^ and is increasingly identified with the use of sophisticated postoperative imaging, such as positron emission tomography-computed tomography (PET-CT).^8-10^ The optimal management of the residual meningioma is an important clinical problem.^3^

A better understanding of growth kinetics could be helpful to influence meningioma management^11,12^; volumetrics can be used to identify the rate of meningioma growth, but there exists a paucity of studies, with none using established progression definitions.^11,13,14^ Identifying the volumetric growth rate of residual meningioma along with prognostic factors for growth could be helpful in clinical management, to stratify patients to receive adjuvant radiotherapy, active surveillance, reoperation, or no treatment.

The primary aim of our study was to identify the volumetric growth rate of residual meningioma and correlate this with factors associated with progression. The secondary objective was to evaluate residual growth trajectory.

## METHODS

### Study Design and Setting

A retrospective analysis of patients with a histological diagnosis of meningioma that underwent surgery and had residual tumor, from 1st January 2004 to 31st August 2020, was performed. This study was conducted at the Walton Centre NHS Foundation Trust in Liverpool, the United Kingdom, a tertiary neurosurgery center with a catchment population of 3.5 million people. Eligible patients had the following: 1. histological diagnosis of meningioma World Health Organization (WHO) grade 1, 2, or 3; 2. surgical resection; and 3. residual tumor on postoperative MRI or defined as Simpson 4 or 5 resection. Patients were excluded if they were confirmed to have a radiation-induced meningioma, multiple meningiomas, or previous intervention before surgery (such as fractionated radiotherapy or stereotactic radiosurgery). Data were reported according to the STROBE guidelines.^15^

### Ethics

This study was approved by the Walton Centre NHS Foundation Trust clinical audit group on 19th February, 2020. Because this study was approved under clinical audit, patient consent was not required.

### Definitions

Residual meningioma was classified as intraoperative reporting of residual tumor on the operative record, reporting of a Simpson grade IV or V or subtotal resection (STR), or identification of a residual tumor on postoperative gadolinium–enhanced T1-weighted MRI scans (1.5–3 T), obtained either within 72 hours or ≥3 months but ≤6 months after surgery (to avoid postsurgical artefacts and differentiate progression from STR). Standard of care at our center is for patients to receive a postoperative MRI 3 months after surgery, followed by scans at 6 and 12 months, followed by annual scanning. If a gross total resection (GTR) was initially reported by the operating surgeon, but residual later identified on imaging and confirmed by multidisciplinary team review including the operating surgeon, those patients were included. Meningioma location was defined according to the International Consortium on Meningioma (ICOM) classification.^2^ WHO grade was defined according to the latest classification at the time of surgery (2000, 2007, or 2016). Extent of resection (EOR) was defined by a board-certified operating neurosurgeon and residual on imaging identified by a board-certified neuroradiologist. Adjuvant fractionated radiotherapy (FRT) was defined as received within 6 months of surgery, without evidence of progression. If used after 6 months, it was considered as treatment of progression. Stereotactic radiosurgery (SRS) is not routinely used in the adjuvant setting at our institution.

### Data Sources

Baseline demographic details (age, sex, and performance status) were identified from patient's medical records. Radiological variables (laterality, ICOM location, calcification, sinus invasion, intensity, and bone invasion) were obtained from MRI scans from the Carestream Vue Picture archiving and communications system (PACS), version 12, a radiological scan database. A single neuroradiologist evaluated all images before surgery. Surgical variables such as extent of resection and complications were gathered from the surgeon's operative notes and case records. Details of adjuvant therapy were captured by accessing the local oncology center (Clatterbridge Cancer Centre) clinical records. The final overall clinical outcome (discharged, still under follow-up, or death) was collected from available medical records.

### Radiological Feature and Tumor Volume Measurement

Baseline MRI was independently (CSG/GER/MAM) reassessed for T2-weighted MRI signal intensity, calcification, venous sinus invasion in proximity to major dural venous sinuses (superior sagittal/transverse/sigmoid/cavernous/torcula categorized as separate [≤10 mm], in direct contact with its wall or invading) and residual tumor volume, and an intraclass correlation coefficient (ICC) on a randomly selected sample of 24 patients (sample size determined using the Bland equation)^16^ calculated to assess agreement.

Tumor volume was measured using the PACS semiautomated measuring tool (**Supplemental Digital Content 1**, http://links.lww.com/NEU/D520). Tumor volume was measured through manual segmentation in the axial plane with manual adjustments made in coronal and sagittal reformats. Each tumor volume was measured, both preoperatively and postoperatively, then at each subsequent scan, censored at the point of the last follow-up or additional intervention (surgery, radiotherapy, or SRS). Volume calculation was adjusted for varying slice scan thickness (0.7–4 mm).

### Growth Curve Measurement

A smaller cohort of patients in whom a minimum of 4 postoperative surveillance MRI studies had been performed had growth curves generated. Deviation from standard scheduling was adjusted for each growth curve by recording the time interval between studies. Meningiomas were excluded from growth curve analysis if they underwent any intervention before having 4 postoperative follow-up MRI scans. Growth was plotted on a volume-time curve, and the nonlinear regression growth curve estimation function was used to approximate best curve fit. Meningioma growth was assessed against 6 growth trajectories, identified previously in meningioma.^17-19^

### Statistical Methods

#### Quantitative Analysis

Statistical analysis was performed using R studio version 4.0.2 (ggplot2, survminer, and blandr packages). Continuous variables were analyzed using mean (SD), or median (IQR), dependent on a histogram, normal distribution curve, and Kolmogorov-Smirnov test of normality.^20^

### Volumetric Growth and Progression Definitions

Volumetric growth was determined using a linear mixed-effects model, which included both the random intercept and the slope, with 100 iterations. We measured the absolute growth rate (AGR) and relative growth rate (RGR) in cm^3^. AGR was defined as $=\left(\left(\frac{V2-V1}{time\;\left(months\right)}\;\right)\times 12\right)$ (increase in volume [V] per year), and RGR was defined as $\left(\left(\frac{\frac{V2-V1}{V1}}{time}\left(months\right)\right)\times 12\times 100\right)$ (percentage increase in volume per year). Tumor progression (regrowth) was defined according to the RANO criteria (absolute increase in volume over 40% at any point during the follow-up period).^11^ To compare RANO with a clinically used definition, we included an additional ‘clinical’ progression definition of radiological growth, defined by a consultant neuroradiologist and validated by multidisciplinary team (tumor board) consensus.

### Growth Curve Analysis

For growth curve analysis, R and R^2^ values were derived from each meningioma to assimilate the best curve type. Quartiles were estimated by linear interpolation between neighboring sample values as necessary. Overall values were combined with the median R^2^ value for each meningioma, with the constant included in curve estimation.

### Progression and Survival Analysis

Progression was estimated by Cox proportional hazards analysis, using both RANO and clinical definitions as end points, time from surgery until progression in months as time, and assessed variables associated with progression using univariable and multivariable analysis. Variables with *P* < .1 on univariable analysis were incorporated into the Cox regression model. Factors were considered significant on multivariable analysis if *P* < .05. We investigated the proportional hazards assumption of the regression model using Schoenfeld residual plots.^21^ Overall survival (OS) and progression-free survival (PFS) function was estimated using Kaplan-Meier curves.

## RESULTS

**FIGURE 1.**
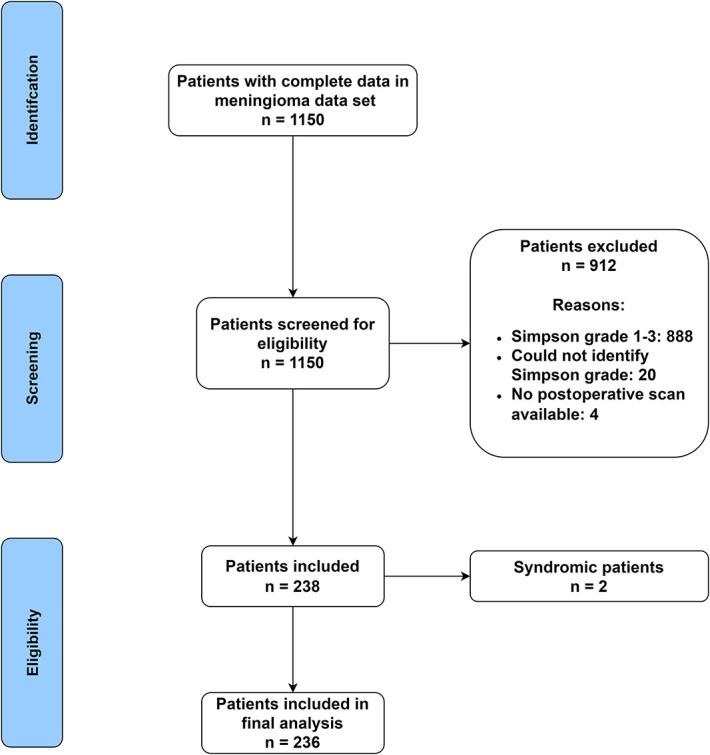
Patient selection process.

Of a total of 728 patients with meningioma who underwent surgery, 236 patients met the inclusion criteria (Figure 1)—a residual meningioma incidence of 32.4%. One hundred and eighty patients (76.3%) had STR/Simpson grade IV or V identified by the operating surgeon, while 56 (23.7%) had residual identified later on postoperative imaging despite an initial impression of complete resection at the time of surgery. One hundred forty four patients (61.0%) had a postoperative MRI at 3 months after surgery.

### Clinical Characteristics

Table 1 summarizes the clinical and demographic characteristics of the cohort. In 24 patients (86.4%), the indication for initial surgery was to reduce symptom burden, in 17 (7.2%) due to patient preference on discovery of an incidental tumor, and in 15 patients (6.4%), it was due to radiological progression of a previously monitored incidental meningioma. There were 195 WHO grade 1 (82.6%), 40 WHO grade 2 (16.9%), and 1 WHO grade 3 meningioma. Ki-67 index was available in 20 patients (8.5%), with a median value of 7.0 (IQR 4.3–11.3).

**TABLE 1.** Baseline Demographics and Clinical Characteristics

Characteristic	Category	N (%)
Age	Mean (SD)	56.3 (13.7)
	<40	29 (12.3)
	40–49	47 (19.9)
	50–59	55 (23.3)
	60–69	61 (25.8)
	70–79	38 (16.1)
	≥80	6 (2.5)
Sex	Male	62 (26.3)
	Female	174 (73.7)
Ethnicity	White British	220 (93.2)
	White—“Others”	6 (2.5)
	White—“European”	2 (0.8)
	Indian	2 (0.8)
	Chinese	2 (0.8)
	Asian—other “Cantonese”	1 (0.4)
	Arabic	1 (0.4)
	White—other “South American”	1 (0.4)
	Unknown	1 (0.4)
Pregnancy/HRT	No	233 (98.7)
	Yes	3 (1.3)
Incidental	Yes	32 (13.6)
	No	204 (86.4)
Symptoms	Headache	65
	Seizures	41
	CN deficit(s)	77
	CN 2	62
	Other CN deficit	19
	Limb weakness	30
	Limb sensory disturbance	12
	Altered GCS	12
	Cognitive deficit	22
	Ataxia	17
WHO Performance status (preoperative)	Median (IQR)	0 (0–1)
	0–1	190 (80.5)
	2–4	46 (19.5)
ACCI (preoperative)	Median (IQR)	2 (1–3)
	0–2	160 (66.4)
	3–5	62 (26.4)
	>5	17 (7.2)

ACCI, age-adjusted Charlson Comorbidity Index; CN, cranial nerve; GCS, Glasgow Coma Scale; HRT, hormone replacement therapy.

### Radiological Characteristics

A summary of radiological characteristics is summarized in Table 2. The median preoperative tumor volume was 34.0 cm^3^ (IQR 16.0–63.0, range 0.2–276.0).

**TABLE 2.** Radiological Characteristics of the Cohort

Characteristic	Category	N (%)
Tumor laterality	Left	94 (39.8)
	Right	96 (40.7)
	Midline	46 (19.5)
Skull base	Yes	140 (59.3)
	No	96 (40.7)
Calcification	Absent	139 (66.5)
	Partial	45 (21.5)
	Diffuse	25 (12.0)
Tumor signal intensity	Hyperintense	120 (63.5)
	Isointense	50 (26.5)
	Hypointense	19 (10.0)
Peritumoral edema	Yes	120 (61.5)
	No	75 (38.5)
Peritumoral edema relative to tumor volume (%)	0–5	14 (12.1)
	6–33	26 (34.5)
	34–66	17 (14.7)
	67–100	12 (10.3)
	>100%	47 (40.5)
Edema volume (cm^3^)	Median (IQR)	39.1 (6.5–85.6)
Edema grade	1	12 (10.3)
	2	35 (30.2)
	3	69 (59.5)
Edema index	Median (IQR)	0.7 (0.1–1.7)
Bone invasion	Yes	67 (33.2)
	No	135 (66.8)
Hyperostosis	Yes	59 (29.2)
	No	143 (70.8)
Sinus invasion	Separate	103 (48.4)
	Direct contact	35 (16.4)
	Invading	75 (35.2)
Compressing critical neurovascular structures	Yes	83 (38.6)
	No	132 (61.4)
Preoperative tumor volume (cm^3^)	Median (IQR)	34.0 (16.0–63.0)
Preoperative tumor diameter (mm)	Median (IQR)	22.7 (10.6–42.0)

### Surgical, Adjuvant Treatment and Follow-up

A summary of the surgical and adjuvant treatments of the cohort are summarized in Table 3. The median percentage tumor resection was 92.0% (IQR 77.5%–97.5%), and the median residual tumor volume was 2.0 cm^3^ (IQR 0.8–5.2). Thirty-one patients (13.1%) received adjuvant FRT. The mean number of follow-up scans per patient was 8.5 (SD 3.9, range 1–24). The median follow-up time after surgery was 64.4 months (IQR 41.7–103.5).

**TABLE 3.** Surgical and Adjuvant Treatments of the Cohort

Characteristic	Category	N (%)
Time to surgery (mo)	Median (IQR)	1.4 (0.5–4.4)
WHO grade	1	195 (82.6)
	2	40 (16.9)
	3	1 (0.4)
Ki-67 index	Median (IQR)	7.0 (4.3–11.3)
Residual tumor volume (cm^3^)	Median (IQR)	2.0 (0.8–5.2)
Percentage of original tumor resected (%)	Median (IQR)	92.1 (77.5–97.5)
Percentage of original tumor remaining (%)	Median (IQR)	7.9 (2.5–22.5)
Additional treatments	No treatment	156 (66.1)
	FRT	68 (28.8)
	SRS	12 (5.1)
Time to FRT (months)	Median (IQR)	10.9 (4.0–44.9)
Adjuvant^a^ FRT?	Yes	31 (13.1)
	No	205 (86.9)
Adjuvant FRT dose (Gy)	54	29 (80.6)
	60	7 (19.4)
SRS	Yes	12 (5.1)
	No	224 (94.9)
SRS dose (Gy)	12.5	3 (25.0)
	15.0	9 (75.0)
Time to SRS (mo)	Median (IQR)	41.3 (15.0–55.2)

a Adjuvant FRT defined as patient receiving FRT within 6 months of the original surgery.

### Volumetric Growth and Progression

**FIGURE 2.**
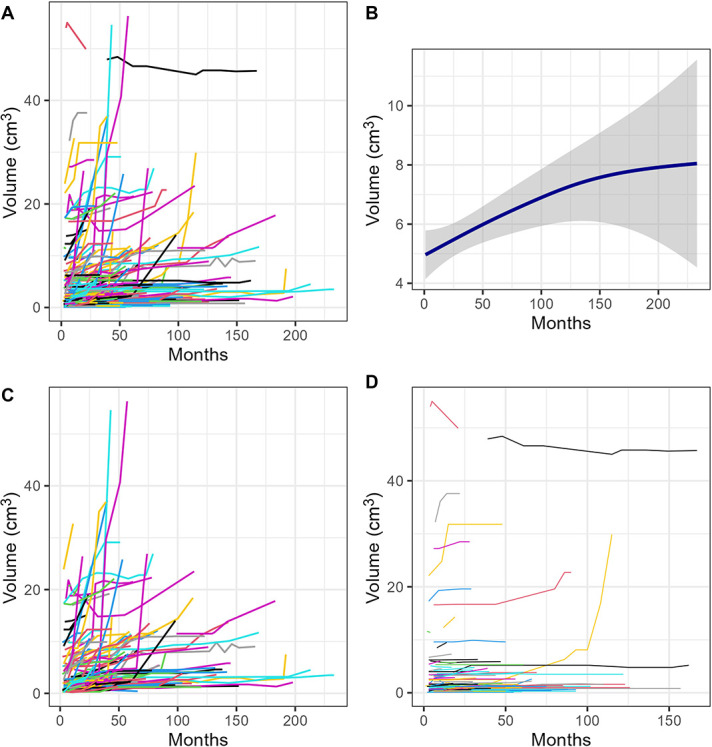
Volume-time growth plots demonstrating **A**, all volumetric growth of meningioma in the study, **B**, smooth conditional means plot demonstrating overall residual tumor growth (with shading representing 95% confidence intervals), **C**, meningiomas that progressed according to the RANO criteria, and **D**, meningiomas that did not progress according to the RANO criteria. RANO, Response Assessment in Neuro-Oncology

Table 4 summarizes the volumetric growth rates. The median annual relative growth rate and absolute growth rate were 4.3% (IQR 1.4%–14.7%) and 0.11 cm^3^/year, respectively. Growth plots of all residual meningioma are shown in Figure 2. One hundred and thirty-two tumors (56.0%)progressed according to the RANO criteria, and 83 (35.2%) had radiological progression defined by the tumor board. Of these, 13 (5.5%) demonstrated clinical progression. The most common symptoms of progression included reduction in visual fields (n = 6) and new-onset headache caused by meningioma (n = 4).

**TABLE 4.** Volumetric Growth and Progression Observed Among the Cohort of 236 Residual Meningiomas

Growth characteristic	Category	N (%)
Absolute growth (cm^3^)	Median (IQR)	2.2 (0.6–12.3)
AGR/year (cm^3^)	Median (IQR)	0.11 (0.03–0.68)
Relative growth (%)	Median (IQR)	82.5 (26.9–284.0)
RGR/year (%)	Median (IQR)	4.3 (1.4–14.7)
Progression as per the RANO criteria	Yes	132 (55.9)
	No	97 (41.1)
Radiological progression as per tumor board	Yes	83 (35.2)
	No	153 (64.8)
Clinical progression	Yes	13 (5.5)
	No	223 (94.5)
Symptoms of progression	Visual field defect progression	6 (42.9)
	Headache	4 (28.6)
	Sensory disturbance	2 (14.3)
	Lump reappearance	1 (7.1)
	Fatigue	1 (7.1)
Time to progression (mo)	Median (IQR)	45.5 (28.4–76.8)

AGR, absolute growth rate; RGR, relative growth rate; RANO, Response Assessment in Neuro-Oncology.

### Progression and Survival Analysis

**FIGURE 3.**
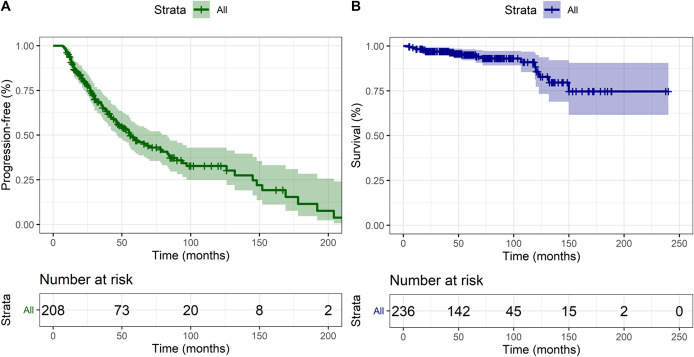
Kaplan-Meier curve demonstrating **A**, progression-free survival and **B**, overall survival in the cohort.

**FIGURE 4.**
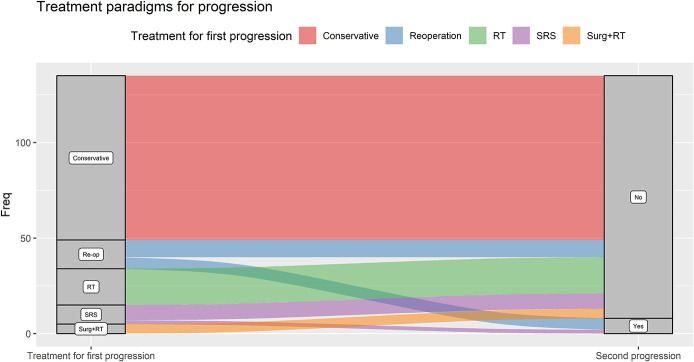
Alluvial plot outlining different treatment paradigms for meningiomas that progressed according to the RANO criteria. RANO, Response Assessment in Neuro-Oncology; SRS, stereotactic radiosurgery.

Time to progression is shown in Figure 3. Eighty-six patients (65.1%) were managed conservatively for their progression (Figure 4). Nineteen (14.4%) were treated with FRT, 15 (11.4%) with repeat surgery alone, 10 (7.6%) with SRS, and 5 (3.8%) with surgery plus adjuvant FRT. Of these 49 patients, 8 (16.3%) progressed further (1 managed conservatively, 3 with FRT, 3 repeat surgery, and 1 with SRS). One patient progressed after a third surgery and was treated with SRS, with no further progression at the last follow-up.

Cox regression models were performed to estimate the unadjusted hazard ratios. In the multivariable model, the variables associated with RANO-defined progression were skull base location (HR 1.60 [95% CI 1.02–2.50], *P* = .042) and Ki-67 index (HR 3.43 [95% CI 1.19–9.90], *P* = .023) (Table 5). The variables associated with clinically defined progression were symptomatic presentation (HR 3.39 [95% CI 1.06–10.81], *P* = .040), WHO grade 2 (HR 1.89 [95% CI 1.10–3.23], *P* = .021), and Ki-67 index (HR 3.41 [95% CI 1.06–11.01], *P* = .040).

**TABLE 5.** Univariable and Multivariable Cox Regression Analysis of Variables Associated With RANO-defined and Radiological Progression

RANO						
	Univariable			Multivariable		
Risk factor	HR	95% CI	*P* value	HR	95% CI	*P* value
Age	1.01	0.99–1.02	.454	—	—	—
Ethnicity (White—Others)	10.88	1.45–81.59	**.020**	1.05	0.78–1.41	.761
Female sex	0.86	0.54–1.37	.521	—	—	—
Pregnancy/HRT	0.82	0.11–5.68	.815	—	—	—
Presentation with symptoms	1.30	0.77–2.20	.322	—	—	—
T2 hyperintensity	0.72	0.47–1.10	.129	—	—	—
Any edema	0.98	0.63–1.52	.910	—	—	—
Edema (cm^3^)	1.00	0.99–1.00	.630	—	—	—
Bone invasion	0.80	0.51–1.25	.324	—	—	—
Hyperostosis	0.80	0.51–1.27	.348	—	—	—
Any calcification	1.52	1.01–2.31	**.047**	1.44	0.95–2.17	.086
Sinus invasion	0.75	0.50–1.13	.167	—	—	—
Compressing a critical neurovascular structure	0.90	0.59–1.37	.628	—	—	—
Skull base location	1.61	1.08–2.41	**.020**	1.60	1.02–2.50	**.042**
Preoperative tumor volume	1.00	0.99–1.00	.648	—	—	—
WHO grade (2)	0.97	0.54–1.73	.965	—	—	—
Ki-67	3.33	1.28–8.67	**.014**	3.43	1.19–9.90	**.023**
Residual tumor volume	1.00	0.98–1.02	.981	—	—	—
Percentage of original tumor remaining	1.00	0.99–1.00	.479	—	—	—
Adjuvant FRT	1.36	0.76–2.44	.299	—	—	—

Radiological—tumor board							
	Univariable			Multivariable			
Risk factor	HR	95% CI	*P* value		HR	95% CI	*P* value
Age	1.00	0.98–1.02	.936		—	—	-
Ethnicity (White–Others)	7.32	0.99–54.07	**.051**		7.52	1.01–55.79	.051
Female sex	0.95	0.57–1.59	.840		—	—	—
Pregnancy/HRT	0.48	0.01–96.71	.435		—	—	—
Presentation with symptoms	3.68	1.16–11.70	**.028**		3.39	1.06–10.81	**.040**
T2 hyperintensity	1.23	0.72–2.10	.446		—	—	—
Any edema	1.16	0.69–1.93	.581		—	—	—
Edema (cm^3^)	1.00	1.00–1.01	.701		—	—	—
Bone invasion	1.02	0.62–1.66	.951		—	—	—
Hyperostosis	1.12	0.67–1.87	.663		—	—	—
Any calcification	0.67	0.39–1.14	.138		—	—	—
Sinus invasion	0.83	0.52–1.32	.419		—	—	—
Compressing a critical neurovascular structure	0.96	0.60–1.54	.860		—	—	—
Skull base location	1.15	0.73–1.83	.549		—	—	—
Preoperative tumor volume	1.00	1.00–1.01	.590		—	—	—
WHO grade (2)	2.07	1.21–3.51	**.008**		1.89	1.10–3.23	**.021**
Ki-67	3.31	1.22–8.99	**.019**		3.41	1.06–11.01	**.040**
Residual tumor volume	0.99	0.96–1.01	.377		—	—	—
Percentage of original tumor remaining	1.00	0.99–1.01	.940		—	—	—
Adjuvant FRT	1.10	0.58–2.09	.767		—	—	—

HRT, hormone replacement therapy; RANO, Response Assessment in Neuro-Oncology.

Bold indicates *P*<0.05.

### Data Validity and Model Assumptions

Both interobserver and intraobserver variability of radiological factors reached at least a good level of agreement (**Supplemental Digital Content 1**, http://links.lww.com/NEU/D520 and **Supplemental Digital Content 2**, http://links.lww.com/NEU/D521). The proportional hazards assumption of the model based on Schoenfeld residuals are shown in **Supplementary Digital content 1**, http://links.lww.com/NEU/D520—the proportional hazards assumption was not violated.

### Growth Curve Estimation

**FIGURE 5.**
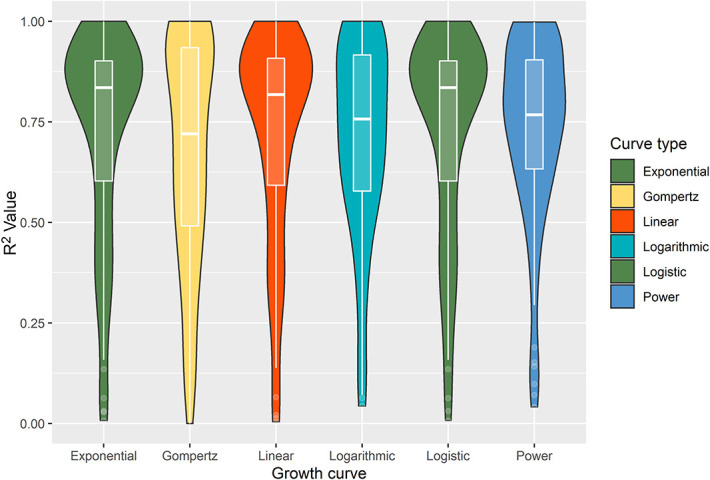
Violin plot (with internal boxplot) of the overall R^2^ values for all meningioma growth curves, stratified by the type of growth curve model. The wider the sections for each “violin,” the more meningioma R^2^ values are located around this. The exponential and logistic models (green) displayed the highest median R^2^ values overall.

Of 236 patients included, 96 had 4 follow-up scans available before intervention to analyze the growth rates (**Supplemental Digital Content 2**, http://links.lww.com/NEU/D521). The violin plots (Figure 5) showed the best curve for estimation of all meningiomas were the logistic and exponential curves (median R^2^ value 0.84 [IQR 0.60–0.90]) and for those receiving intervention before 4 postoperative follow-up scans (n = 14, median R^2^ value 0.87 [IQR 0.68–0.95]).

## DISCUSSION

Residual meningioma is observed in one-third of patients undergoing surgery.^4,5,14^ In this study, we report the largest series of growth curve analysis of residual meningioma and the first of its kind, which demonstrated a low absolute and relative growth rate. We measured volumetric growth using manual segmentation to ensure precise measurements^22^ and used established progression definitions according to RANO, not yet used in published volumetric studies.^23,24^ The absolute (0.11 cm^3^/year) and relative (4.3%/year) growth rates are consistent with those reported in the literature.^14^ In untreated meningiomas, the best growth curve overall was postulated to be the Gompertz curve,^18,19^ but in our study, the most accurate curves were exponential and logistic, with Gompertz demonstrating the smallest median R^2^ value overall.

Because most meningioma are postulated to occur many years before causing symptoms, a meningioma that requires surgery may have already passed the inflection point in its growth trajectory, with subsequently increased likelihood of exponential growth, in comparison with incidental or sporadic meningioma.^17,19,25^ It has been hypothesized in preclinical oncology studies that increased tumor size correlates with an increasing likelihood of cells entering a quiescent phase and subsequently re-enter the proliferation cycle; however, this remains to be investigated in meningioma-specific studies.^26^ The potential change from sigmoidal to exponential growth exhibited in residual meningioma has also not been explored.^27^ The role of the resection cavity increasing space, the interaction within the tumor microenvironment, and patient variables such as age and sex in influencing this growth also remain to be elucidated.^18^

Owing to the relatively indolent nature of most meningioma, ‘active monitoring’ of the residual tumor was used for most patients, and this approach is supported by the finding that very few patients had clinical progression associated with radiological growth.^28,29^ Elevated Ki-67 index has previously been noted to predict time to recurrence in a prospective cohort of surgically treated meningioma consisting of both GTR and STR cases^30^; however, this was only available for 20 patients within our study, likely representing a highly selected population. Radiation-induced meningiomas have been noted to display increased volumetric growth rates, in comparison with sporadic meningiomas, hence why they were not included in our study.^31^ In a study of 141 patients with WHO grade 1 meningioma who had not received adjuvant FRT, preoperative residual volume, location, and ethnicity were identified to correlate with growth.^14^

It is notable that previously identified prognostic factors^32,33^ (residual volume, WHO grade, and signal hyperintensity) were not identified as being associated with progression in our study. This could be due to the lack of a multivariable analysis in previous studies^32,33^ or the fact that in our study we used RANO progression definition, compared with other studies which defined progression according to a smaller volume increase.^14^

There has been considerable interest in integrating a combination of imaging, clinical, and biological data to personalize management of patients with meningioma—including histopathological features such as Ki-67 index and brain invasion, growth rates, and methylation classification.^30,34,35^ In addition, mutation status, in particular *NF2* in driving high-grade growth and *TRAF7*, *KLF4*, *ATK1*, *SMARCE1*, and *SMO* in reducing growth, combined with number and area of copy number alterations, remain highly promising areas of exploration.^36-38^ Despite this, a long-term follow-up demonstrates that even after complete resection, the risk of regrowth and late recurrence continues.^39,40^ Therefore, the optimal management of patients with residual meningioma is a continued area of clinical uncertainty.^41^ The European Association of Neuro-Oncology (EANO) guidelines advocate adjuvant FRT for WHO grade 1 meningioma undergoing STR.^3^ Our results suggest that patients have a low rate of clinical progression and those that progress could be managed with conservative management in the early stages. In the future, the volumetric growth rate, combined with emerging molecular data, may shape and guide this treatment decision.

### Limitations

This study has several limitations. First, the population is a retrospective cohort of patients from a single center. As such, there was heterogeneity in the MRI data with studies performed on a variety of scanners, with a range of field strengths and using slightly different imaging protocols. Although the PACS interpolated volume measurement is an approved clinical method of measuring volumes, there is potential for underestimation/overestimation of volumes when using noncontiguous acquisitions, leading to an increased proportion of tumors displaying exponential growth. Second, the median follow-up time was limited to 5 years after surgery—a more prolonged follow-up time may reveal additional progression events, as the literature now suggests that risk of recurrence continues beyond 10 years after initial surgery/treatment.^42,43^ Third, although only 96 patients had sufficient MRI scans available before intervention for the volumetric analysis of growth curves, this is still the largest analysis of growth curves for any meningioma type reported in the literature and the only one reporting on residual tumor. Fourth, owing to changes in the WHO classification over the study period, some grade 1 meningiomas may be reclassified as grade 2. This may account for the lack of difference in growth rates between WHO grade 1 and 2 meningiomas. Finally, the RANO-defined progression criteria could be considered less applicable to everyday neurosurgical practice. Hence, we also incorporated a multidisciplinary definition of progression.

## CONCLUSION

There is no agreed standard of care for the management of residual meningioma. Our study has shown that the volumetric growth rate of residual meningioma is low, and only 5% of patients develop neurological symptoms indicative of clinical progression at follow-up. A period of active MRI monitoring is therefore recommended for most cases.

## Supplementary Material

SUPPLEMENTARY MATERIAL

## Supplemental Digital Content

**Supplemental Digital Content 1. Figure 1.** Figure demonstrating the PACS semiautomated measuring tool for volumetric analysis (Images 1–6). Manual tracing is performed for each axial slice of tumor and the volume calculated (larger tracing line used for illustrative purposes). **Supplemental Digital Content 1. Figure 2.** Figure demonstrating the PACS semiautomated measuring tool for residual tumor volume measurement (Images 1–6). Manual tracing is performed for each axial slice of tumor and the volume calculated (larger tracing line used for illustrative purposes). **Supplemental Digital Content 1. Figure 3.** Heat map demonstrating ICOM location and residual tumor volume, with density representing number of cases. **Supplemental Digital Content 1. Figure 4.** Bland-Altman plot with mean residual volume intercept (1.15 cm^3^) for interobserver variability (A) and intraobserver variability (B). All points are within the 95% CI indicating a good level of agreement. **Supplemental Digital Content 1. Figure 5.** Schoenfeld residual plot for each of the covariates. The solid line is a smoothing spline fit to the plot, with the dashed lines representing a ± 2-standard error band around the fit. None of the plots demonstrated a regular pattern with time, and tests were all not statistically significant. **Supplemental Digital Content 1. Figure 6.** Volume-time growth plot demonstrating meningioma volumetric growth, stratified by those that received adjuvant FRT (black). **Supplemental Digital Content 1. Figure 7.** Kaplan-Meier curve demonstrating progression-free survival (PFS) in the cohort, stratified by adjuvant FRT. **Supplemental Digital Content 1. Figure 8.** Violin plot (with internal boxplot) of the overall R^2^ values for meningioma that received early intervention (fractionated radiotherapy, repeat surgery, and stereotactic radiosurgery), stratified by type of growth curve model. The wider the sections for each ‘violin,’ the more meningioma R^2^ values are located around this. The exponential (red) and logistic models (blue) displayed the highest median R^2^ values overall. **Supplemental Digital Content 2. Table 1.** Surgical and adjuvant treatments of the cohort. **Supplemental Digital Content 2. Table 2.** Univariable analysis of variables associated with progression. **Supplemental Digital Content 2. Table 3.** Weighted Kappa values assessing the interobserver and intraobserver variability among variables. **Supplemental Digital Content 2. Table 4.** Coefficients of determination (all meningiomas, n = 96). **Supplemental Digital Content 2. Table 5.** Coefficients of determination (early intervention group, n = 14).
