# Supplementary material for: Volumetric Growth and Growth Curve Analysis of Residual Intracranial Meningioma
Source: Neurosurgery. 2022 Dec 14;92(4):734–44. doi: 10.1227/neu.0000000000002268 (PMC9988310; doi:10.1227/neu.0000000000002268)
Supplement: SUPPLEMENTARY MATERIAL [file neu-92-734-s001.docx]

Supplemental Digital Content 2. Table 1.

| Supplemental Digital Content 2. Table 1. Surgical and adjuvant treatments of the cohort. | | |
| --- | --- | --- |
| Characteristic |  | **N (%)** |
| Time to surgery (months) | Median (IQR) | 1.4 (0.5-4.4) |
| WHO grade | 1 | 195 (82.6) |
|  | 2 | 40 (16.9) |
|  | 3 | 1 (0.4) |
| Ki-67 index | Median (IQR) | 7.0 (4.3-11.3) |
| Residual tumour volume (cm^3^) | Median (IQR) | 2.0 (0.8-5.2) |
| Percentage of original tumour resected (%) | Median (IQR) | 92.1 (77.5-97.5) |
| Percentage of original tumour remaining (%) | Median (IQR) | 7.9 (2.5-22.5) |
| Additional treatments | No treatment | 156 (66.1) |
|  | *f*RT | 68 (28.8) |
|  | SRS | 12 (5.1) |
| Time to *f*RT (months) | Median (IQR) | 10.9 (4.0-44.9) |
| Adjuvant* *f*RT? | Yes | 36 (15.3) |
|  | No | 200 (84.7) |
| *Adjuvant *f*RT defined as patient receiving *f*RT within 6 months of the original surgery. | | |

Supplemental Digital Content 2. Table 2.

| Supplemental Digital Content 2. Table 2. Univariable analysis of variables associated with progression. | | | |
| --- | --- | --- | --- |
| Risk factor | **Hazard ratio (HR)** | **95% CI** | **P value*** |
| Age | 1.01 | 0.99-1.02 | 0.454 |
| Ethnicity (White- other) | 10.88 | 1.45-81.59 | **0.020*** |
| Radiation-Induced | 10.46 | 1.35-80.93 | **0.025*** |
| Female sex | 0.86 | 0.54-1.37 | 0.521 |
| Pregnancy/HRT | 0.82 | 0.11-5.68 | 0.815 |
| Presentation with symptoms | 1.30 | 0.77-2.20 | 0.322 |
| T2 hyperintensity | 0.72 | 0.47-1.10 | 0.129 |
| Any Oedema | 0.98 | 0.63-1.52 | 0.910 |
| Oedema (cm^3^) | 1.00 | 0.99-1.00 | 0.630 |
| Bone invasion | 0.80 | 0.51-1.25 | 0.324 |
| Hyperostosis | 0.80 | 0.51-1.27 | 0.348 |
| Any calcification | 1.50 | 0.99-2.26 | **0.056*** |
| Sinus invasion | 0.75 | 0.50-1.13 | 0.167 |
| Compressing a critical neurovascular structure | 0.90 | 0.59-1.37 | 0.628 |
| Skull base location | 1.51 | 1.02-2.23 | **0.039*** |
| Pre-operative tumour volume | 1.00 | 0.99-1.00 | 0.648 |
| WHO grade (2) | 0.97 | 0.54-1.73 | 0.965 |
| Ki-67 | 3.33 | 1.28-8.67 | **0.014*** |
| Residual tumour volume | 1.00 | 0.98-1.02 | 0.981 |
| % of original tumour remaining | 1.00 | 0.99-1.00 | 0.479 |
| Adjuvant *f*RT | 1.86 | 1.11-3.10 | **0.018*** |

Supplemental Digital Content 2. Table 3.

| Supplemental Digital Content 2. Table 3. Weighted Kappa values assessing the inter- and intra-observer variability among variables. | | | |
| --- | --- | --- | --- |
|  | **Weighted Kappa (95% CI)** | | |
| Parameter | Inter-observer variability | | Intra-observer variability |
| Calcification | 0.747 (0.416-1.000) | | 0.908 (0.731-1.000) |
| Tumour signal intensity | 0.714 (0.424-1.000) | | 0.822 (0.587-1.000) |
| Residual tumour volume | 0.984 (0.963-0.993) | | 0.988 (0.972-0.995) |
| Sinus invasion | 0.673 (0.400-0.949) | | 0.660 (0.360-0.959) |
|  | | **ICC (95% CI)** | |
|  | Inter-rater variability | | Intra-rater variability |
| Residual tumour volume | 0.984 (0.963-0.993) | | 0.988 (0.972-0.995) |

Supplemental Digital Content 2. Table 4.

| Supplemental Digital Content 2. Table 4. Coefficients of determination (all meningiomas, n = 96) | | | |
| --- | --- | --- | --- |
| Regression model | **Percentile** | | |
|  | 25^th^ | 50^th^ (median) | 75th |
| Linear | 0.57 | 0.82 | 0.91 |
| Logarithmic | 0.58 | 0.76 | 0.92 |
| Power | 0.63 | 0.77 | 0.92 |
| Gompertz | 0.49 | 0.72 | 0.94 |
| Exponential | 0.60 | **0.84** | 0.90 |
| Logistic | 0.60 | **0.84** | 0.90 |
| Highest curve estimations | | **N (%)** | |
| Exponential and Logistic | | 37 (38.5) | |
| Gompertz | | 35 (36.5) | |
| Tie (linear, exponential and logistic) | | 7 (7.3) | |
| Power | | 7 (7.3) | |
| Logarithmic | | 6 (6.3) | |
| Linear | | 4 (4.2) | |

Supplemental Digital Content 2. Table 5.

| Supplemental Digital Content 2. Table 5. Coefficients of determination (early intervention group, n = 14). | | | |
| --- | --- | --- | --- |
| Regression model | **Percentile** | | |
|  | 25^th^ | 50^th^ (median) | 75th |
| Linear | 0.74 | 0.85 | 0.93 |
| Logarithmic | 0.74 | 0.79 | 0.95 |
| Power | 0.76 | 0.84 | 0.94 |
| Gompertz | 0.66 | 0.86 | 0.98 |
| Exponential | 0.68 | **0.87** | 0.95 |
| Logistic | 0.68 | **0.87** | 0.95 |
| Highest curve estimations | | **N (%)** | |
| Exponential and Logistic | | 6 (42.9) | |
| Gompertz | | 6 (42.9) | |
| Tie (linear, exponential and logistic) | | 1 (7.1) | |
| Power | | 1 (7.1) | |
| Logarithmic | | 0 (0.0) | |
| Linear | | 0 (0.0) | |
